# Oxidative and Cellular Stress Markers in Postmenopause Women with Diabetes: The Impact of Years of Menopause

**DOI:** 10.1155/2021/3314871

**Published:** 2021-09-16

**Authors:** Carolain Felipin Vincensi Anklam, Yana Picinin Sandri Lissarassa, Analú Bender dos Santos, Lílian Corrêa Costa-Beber, Lucas Machado Sulzbacher, Pauline Brendler Goettems-Fiorin, Thiago Gomes Heck, Matias Nunes Frizzo, Mirna Stela Ludwig

**Affiliations:** ^1^Research Group in Physiology, Department of Life Sciences, Regional University of Northwestern Rio Grande Do Sul State (UNIJUI), Rua do Comércio, 3000 Bairro Universitário Ijuí RS, Brazil 98700-000; ^2^Postgraduate Program in Integral Attention to Health (PPGAIS-UNIJUÍ/UNICRUZ), Ijuí, RS, Brazil; ^3^Postgraduate Program in Mathematical and Computational Modeling (PPGMMC-UNIJUÍ), Ijuí, RS, Brazil

## Abstract

Women live approximately one-third of their lives in postmenopause. Among postmenopausal women, type 2 diabetes mellitus (DM2) is one of the most prevalent chronic diseases. These conditions promote alterations in the oxidative, metabolic, and immune-inflammatory profiles marked by higher extracellular 72 kDa-heat shock protein (eHSP72). Here, we investigated whether the time of menopause is associated with oxidative cellular stress marker levels in postmenopausal women with DM2. Sixty-four women were recruited (56.7 ± 12.6 years old) in the pre- (*n* = 22) and postmenopause (*n* = 42) period, with (*n* = 19) or without DM2 (*n* = 45), and a fasting blood collection was made for the evaluation of metabolic, oxidative, and inflammatory markers. We found that menopause and DM2 influenced metabolic and oxidative parameters and presented synergistic effects on the plasma lipoperoxidation levels. Also, postmenopausal women had the highest eHSP72 concentration levels associated with the years in postmenopause. We conclude that the time of menopause impacts the markers of cellular stress and increases the risk of oxidative stress, mainly when it is associated with DM2.

## 1. Introduction

Population aging observed in most countries has led women to live one-third of their lives in the postmenopausal condition [1]. Menopause marks the end of the reproductive life and is characterized by the decline of 17*β*-estradiol levels. It predisposes to oxidative stress [2], vasomotor symptoms [3], osteoporosis, and chronic diseases, such as obesity and type II diabetes mellitus (DM2) [4].

DM2 is one of the most prevalent chronic diseases in postmenopausal women and is characterized by metabolic disorders, as well as a chronic low-grade inflammatory and oxidative disbalance. Oxidative stress appears to be central in the DM2 progression since the lipoperoxidation increases according to the disease severity [5]. Hence, the global (metabolic, oxidative, and inflammatory) impairment that characterizes DM2 can increase the susceptibility to complications when associated with low estrogen levels [6].

Both DM2 and hypoestrogenism imply changes in the cellular stress response, mainly the 72 kDa-heat shock proteins (HSP72) [7]. The anti-inflammatory, antiapoptotic, and cytoprotective roles of HSP72 in the intracellular medium contrast with its effects in the extracellular medium [8]. HSP72 is released to the extracellular milieu (eHSP72), and, when circulating, it can bind to toll-like receptors and act in the proinflammatory and damage signaling [9]. Therefore, managing its plasmatic concentration may represent a potential therapeutic target [10, 11].

It was previously reported that the circulating levels of HSP72 are associated with cardiovascular risk in postmenopausal women with diabetes [12]. In this sense, the role of eHSP72 as predictive in clinical conditions [10, 11], mainly chronic and inflammatory ones, has been investigated [13]. Hence, the eHSP72 plasmatic concentration along the postmenopausal period could help identify the physiopathological relationships between postmenopause and DM2. However, whether it is sensitive to the low-grade inflammation that marks postmenopause plus DM2 and the follow-up of this scenario years after menopause remains unclear. Thus, we hereby evaluated if the time of postmenopause affects the circulating levels of eHSP72 in diabetic women and if it is associated with an oxidative and inflammatory profile.

## 2. Methods

Women participating in care groups in the Family Health Strategies of a town in southern Brazil were recruited and participated in our study. Initially, 73 women were administered face-to-face interviews to obtain sociodemographic information, medical history of chronic diseases, time of amenorrhea, use of medications, and dietary survey. Further, we applied as exclusion criteria the current use of hormonal replacement therapy, cancer, autoimmune, acute infection, nontreated hypertension, smoking, undergoing chemotherapy treatment, and the regular or eventual use of insulin. We ended up with 64 women participating in our study. The calculation of the sample number was done based on the expected difference for the most important variable in this study, the circulating concentration of eHSP72, as in the Nakhjavani et al. [14] study, and indicates a number of 15 subjects per group. We used a statistical power of 95%, with a significance level of 0.05%. It is worth noting that, during the study, the number of samples used for each parameter varied (as expressed in the figure and table legends) due to its technical conditions for the analysis.

The participants were initially divided in two groups: premenopause (*n* = 22) and postmenopause (*n* = 42). Postmenopause was defined as amenorrhea of 12 months, at least, which was confirmed by the estrogen (17*β*-estradiol) levels (premenopause: 55.3 ± 54.7 pg/mL and postmenopause 25.3 ± 18.6 pg/mL). Finally, they were divided in subgroups: premenopause without DM2 (*n* = 15), premenopause with DM2 (*n* = 7), postmenopause without DM2 (*n* = 30), and postmenopause with DM2 (*n* = 12), based on medical diagnosis of DM2 or by fasting glycemia ≥ 126 mg/dL and HbA1c > 6.5% [15]. Based on Nakhjavani et al.'s [14] study, which showed that long-standing diabetes had higher eHSP72 levels than controls, we expect similar biological evidence (difference of eHSP72 levels ~0.58 ± 0.35 ng/mL) to reach a statistical power of 95%, with a significance level of 0.05% when comparing premenopause without DM2 vs. postmenopause with DM2. The study had 95.0% power to detect an effect size of 0.509 using T statistics or better (0.489) using the Z statistic instead of the T statistics.

The study was made in agreement with the Regulatory Guidelines and Norms for Research Involving Humans, according to Resolution of the National Health Council (CNS) n°. 466/2012 and was approved by the Ethics Committee (n° 1.173.158).

### 2.1. Anthropometric Analyses

Bodyweight (kg) was verified using a calibrated scale and the height (cm) and waist circumference (WC), abdominal (AC), and hip circumference (HC), with a standard measuring tape. To analyze WC, we admitted the Brazilian Guidelines of Obesity 2009-2010 and its values of risk for metabolic complications [16]. We also evaluated the waist-to-hip ratio by the direct quotient between waist and hip circumference, classified according to the cut points recommended by World Health Organization [16].

We calculated body mass index (BMI) using the Quetelet equation, BMI (kg/m^2^) = mass (kg)/height (m)^2^  and analyzed it according to the Brazilian Association for the Study of Obesity guidelines [16]. As a complement, we evaluated the adiposity index using the equation $\text{Adiposity Index}=\left[\text{HC }\left(\text{cm}\right)/\text{height }\left(\text{m}\right)\times \sqrt{\text{height }\left(\text{m}\right)}\right]-18$ [17] and the conicity index according to the formula $\text{Conicity Index}=\text{WC }\left(\text{m}\right)/0.109\times \sqrt{\text{weight }\left(\text{kg}\right)/\text{height }\left(\text{m}\right)\;}$.

### 2.2. Blood Collection

Biochemical analyses were performed in blood collected from patients after 10 to 12 hours of fasting. Blood was immediately separated in vacuum tubes with and without ethylenediaminetetraacetic acid (EDTA). Blood with EDTA was used for total blood aliquot and plasma separation. Blood without EDTA was used to obtain serum. Aliquots for plasma and serum were centrifuged for 30 minutes at 3.000 rpm. Total blood was used to measure glycated hemoglobin, erythrocyte sedimentation rate, and leukometry; serum was used to measure E2 levels as well as lipid, glycemic, and hepatic profiles. Plasma samples were frozen in liquid nitrogen with phenyl methyl sulfonyl fluoride (PMSF, Sigma P7626, FW = 174.19 g/mol) (1.74 mg/mL = 100 mM) for subsequent measurement of malondialdehyde (MDA) and eHSP72 levels.

### 2.3. Dosage of 17*β* Estradiol

A quantitative dosage of 17*β*-estradiol (E2) was performed in a serum sample through the automated system ADVIA Centaur XP (Siemens Healthcare Diagnosis) by chemiluminescence methodology with sensitivity and in vitro test limits higher than 20 pg/mL. The results were expressed in pg/mL.

### 2.4. Lipid, Glycemic, and Hepatic Profile

Total cholesterol, HDL, triglycerides levels, and fasting glycemia were analyzed in serum samples by an enzymatic-colorimetric method using Bioclin-Quibasa kits in BS200-Mindray automation. LDL was indirectly verified by the Friedewald equation LDL = CT − HDL − TG/5, where TG/5 represents the cholesterol bound to VLDL-C [18].

Glycated hemoglobin was measured in total blood aliquot by high-performance liquid chromatography (HPLC) and expressed in percentage. The mean glycemia was estimated by the formula (Mean glycemia = 28.7 × HbA1C) − 46.7, in agreement with the recommendations from the Brazilian Society of Diabetes [15]. All other results were expressed in mg/dL.

Hepatic enzymes were measured in serum samples. Alkaline phosphatase (ALF), glutamic-oxaloacetic transaminase (GOT), glutamic-pyruvic transaminase (GPT), and gamma-glutamyl transferase (GGT) were measured by kinetic methods using Biclin-Quibasa kits. The results were expressed in U/L.

### 2.5. Inflammatory Status

Ultrasensitive C-reactive protein was measured in serum samples by turbidimetry. The results were expressed in mg/dL. Erythrocyte sedimentation rate was verified in total blood by Westergren's pipette technique. Leukometry was measured by impedance counting, obtaining the number of total leukocytes/mm^3^.

eHSP72 levels were verified in plasma samples by a highly sensitive enzyme-linked immunosorbent assay. We used an HSPA1A-specific HSP72 ELISA Kit (ENZO Life Sciences, ENZ-KIT-101) according to the manufacture's recommendations. A standard curve was constructed from known dilutions of HSP72 recombinant protein to allow a quantitative assessment of eHSP72 plasma concentration. Quantification was done using a microplate reader (Mindray MR-96A) at 450 nm, and the intra-assay coefficient of variation was identified as being <2%. Results were expressed in *η*g/mL.

### 2.6. Lipoperoxidation

Lipoperoxidation was measured by the MDA levels using the thiobarbituric acid reactive substances method (TBARS) [19]. Briefly, 25 *μ*L of plasma was incubated with 20 *μ*L water, 125 *μ*L thiobarbituric acid (TBA, 0.6%), 5 *μ*L butylated hydroxytoluene (BHT, 10 mM), 300 *μ*L of phosphoric acid (H_3_PO_4_, 1%), and 25 *μ*L sodium dodecyl sulfate (SDS, 8.1%) for 60 min at 100°C. After this, tubes were centrifuged, the supernatant collected, and the absorbance verified in a plate reader (DR-200BS model, Kasuaki, PR, Brazil) at 505 nm. The MDA standard was prepared from 1.1.3.3-Tetramethoxypropane (points from 0.0005–0.016 mg/mL). Results were expressed in mmol MDA/mg of protein.

### 2.7. Statistical Analysis

Initially, we verified the data normality by the Kolmogorov-Smirnoff test. We compared means of E2 and eHSP72 levels between pre-and postmenopausal women using the Student's *t*-test and evaluated the association between menopause and eHSP72 levels by chi-square test.

We further distributed women in groups according to the “time of menopause” and “age” based on the median values of time of postmenopause and age (expressed in years). We compared eHSP72, CRP, and E2 levels between the established groups using one-way ANOVA, followed by Tukey. We also evaluated the effect of interaction between DM2 and menopause in different parameters by using two-way ANOVA, followed by Tukey.

For analyses of the association between anthropometric, metabolic parameters, time of menopause, presence of DM2, and time of DM2 with the eHSP72 levels (dependent variable), we used the univariate and multivariate regression and the Pearson correlation. We adjusted the quadratic regression to be linear.

The data were processed by Graphpad Prism 9.0, and the results were expressed in mean ± standard deviation.

## 3. Results

The mean age of the 64 women was 56.7 ± 12.6 years. The premenopausal women (*n* = 22) presented 43.3 ± 8.0 years and the postmenopausal women (*n* = 42), 63.5 ± 8.3 years. The mean value of abdominal circumference (AC) was higher than 88 cm (value indicating metabolic risk) in all groups, reaching 94% of postmenopausal women and 86% of premenopausal women. About 30% of the women were included in the DM2 group. Of these women, 63% reported hot flushes compared to 42% of the postmenopausal without DM2. Among postmenopausal women, 88% (37) reported using statins and presented LDL levels below 150 mg/dL.

As expected, the fasting glycemia, HbA1c, and MEG were higher for diabetic women, without differing for pre- and postmenopausal women.

LDL was influenced by both risk factors, isolated or associated (Table 1). Postmenopausal women with DM2 presented a lower concentration of LDL compared to postmenopausal women without DM2 (Table 1), and these values were inversely related to the use of statins. DM2 also enhanced triglycerides levels, and this effect was similar between pre- and postmenopausal women. Total cholesterol was influenced by diabetes, without an additional effect of menopause (Table 1).

In general, the hepatic profile was also disturbed by menopause and DM2. The serum levels of GGT were enhanced by the interaction between factors. Premenopausal women with diabetes presented higher levels of GGT than all other groups. In addition, postmenopausal women with diabetes presented higher GGT levels than postmenopausal women without diabetes. The ALF was affected by menopause, and the effect was similar in women with or without DM2. The transaminases were not responsive to the factors (Table 1).

Further, we verified the lipoperoxidation in the plasma by evaluating the MDA levels, and we found that it was influenced by both factors, isolated or associated. Postmenopausal women with DM2 presented higher levels of MDA compared to premenopausal women with or without DM2, as well as compared to postmenopausal women with DM2 (Table 1). Thus, we performed multivariate regression analysis between the MDA levels (independent variable) and the menopause status, women's age, and DM2 diagnosis, and the statistics confirmed an association between MDA levels and DM2 diagnosis (*R*^2^_aj_ 0.27; *p* = 0.0009).

The leukometry analysis showed an influence of menopause and the interaction between both risk factors in a way that postmenopausal women with DM2 presented more leukocytes compared to postmenopausal women without DM2. The erythrocyte sedimentation rate (ESR) was also affected by the interaction between menopause and DM2, although a multiple comparisons analysis did not indicate the difference between groups. Interestingly, the CRP levels were not responsive to menopause status or DM2 condition (Table 1).

**Figure 1:**
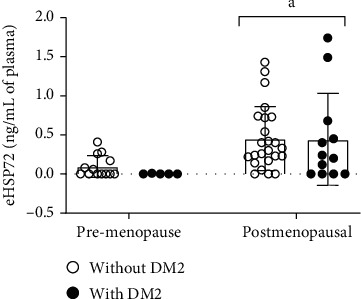
Plasma eHSP72 levels in premenopausal women without DM2 (*n* = 13) and with DM2 (*n* = 05) and postmenopausal women without DM2 (*n* = 24) and with DM2 (*n* = 12). Data were expressed in mean ± standard deviation. Two-way ANOVA, followed by Tukey. Menopause *p* = 0.0026; diabetes *p* = 0.6788; interaction *p* = 0.7374. ^a^vs. premenopause.

Thus, we also analyzed eHSP72 levels as a biomarker of the inflammatory status. The chi-square test showed that 83.3% of the postmenopausal women presented detectable levels of circulating eHSP72, while only 50% of premenopausal women presented detectable levels (*p* = 0.0048). Besides, postmenopausal women (0.4494 ± 0.07804, *n* = 36) presented higher eHSP72 levels compared to premenopausal women (0.07031 ± 0.02936, *n* = 18), and this effect was independent of DM2 diagnosis (Figure 1). We stratified women according to their ages (group 27–59 years old and group 60–83 years old), considering the median age of the postmenopausal women. The eHSP72 levels did not differ among groups (*p* = 0.0717).

Following the comparison between our four initial groups, we implemented a detailed evaluation of the postmenopausal women's conditions. We performed a multivariate regression analysis of eHSP72 levels (dependent variable) and 17-*β* estradiol (E_2_) levels, time of menopause, and DM2 diagnosis, considering only the postmenopausal women. We found that both factors affect the eHSP72 levels (Table 2).

**Figure 2:**
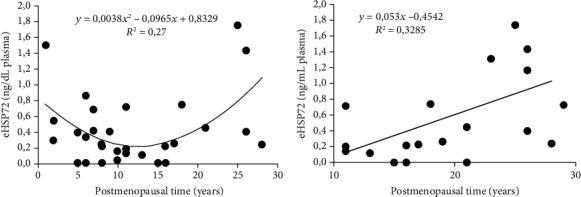
(a) Relation between eHSP72 levels and time of menopause. Positive correlation, linearized by quadratic regression (*n* = 36; *p* = 0.0045). (b) Relation between eHSP72 and the time of menopause in women 12 to 29 years postmenopause. Linear and simple regression, positive correlation (*n* = 17; *p* = 0.0248).

The analysis of the association between eHSP72 and time of menopause reveals a curvilinear relationship between factors, with decreasing values over the first 11 years after menopause and subsequent elevation (Figure 2(a)). The line adjusts by the quadratic regression indicate a positive association between plasma eHSP72 and time of menopause (*R*^2^ 0.23541, *p* = 0.0045) (Figure 2(a)).

Further, we stratified postmenopausal women into two groups according to the median of the time of menopause. We established two groups: one composed of women with one to 11 years postmenopause, the other composed of women 12 to 29 years postmenopause. Thus, we analyzed in each group the correlation between eHSP72 and the time of menopause. We found a linear and positive association between eHSP72 levels and the time of menopause in women with 12 to 29 years of menopause (*R*^2^ 0.33; *p* = 0.0248; *r* = 0.56) (Figure 2(b)).

**Figure 3:**
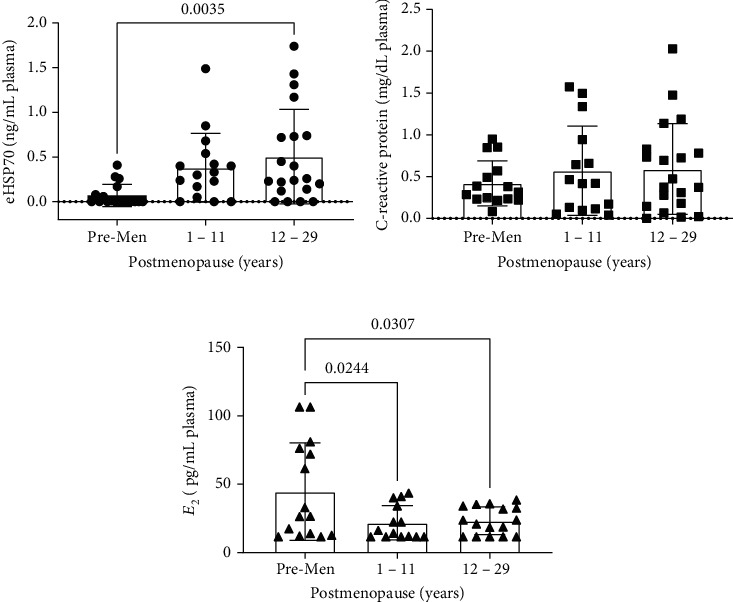
(a) eHSP7; (b) C-reactive protein (CRP); and (c) 17*β*-estradiol (E2) levels, in premenopausal (premen) (*n* = 15–18) and postmenopausal women 1 to 11 years (*n* = 14–16) and 12 to 29 years of postmenopause (*n* = 17–20). One-way ANOVA, followed by Tukey. ^a^vs. premenopausal woman, *p* < 0.05.

Thus, we confirmed these results by comparing the eHSP72 levels between the groups of postmenopausal (group 1–11 years of postmenopause and group 12–29 years of postmenopause) and the premenopausal women. Women 12 to 29 years of postmenopause presented higher eHSP72 levels than premenopausal women (Figure 3(a)). On the other hand, CRP did not differ between groups (Figure 3(b)). As expected, both postmenopausal groups presented lower E2 levels compared to premenopausal women, but it was not affected by the time of menopause (Figure 3(c)).

## 4. Discussion

In our study, postmenopause was marked by increased eHSP72 levels, which were affected by the E2 levels, DM2, and, most important, by the time of menopause. In addition, we found that menopause combined with DM2 predisposed to plasma oxidative damage and affected hepatic functions. Besides, postmenopause represented an additional risk factor for lipid homeostasis when not followed by pharmaceutical treatment. Therefore, our data support the hypothesis that eHSP72 can be a potential biomarker of the immune and inflammatory status in postmenopause conditions.

The complexity of the menopause period reflects in anthropometric, metabolic, and endocrine changes [20]. The gradual downfall of E2 production by ovaries is usually followed by changes in body composition, such as the gradual decrease of muscle mass and the increase of adiposity, mainly centrally disposed [21]. Not surprisingly, 94% of the postmenopausal and 86% of premenopausal women from our study had abdominal circumference superior to 88 cm, which represents metabolic risk.

Obesity is an important risk factor for insulin resistance [22], although not necessary since hypoestrogenism per se influences glycemic homeostasis [23]. In our study, as expected, diabetic women presented higher fasting glycemia and HbA1c in pre- and postmenopause, without differing between menopausal status. Thus, we analyzed only healthy women (without DM2, normotensives, not currently using medications), and we found that HbA1 was higher in postmenopausal. In addition, 49% of “healthy” postmenopausal women were at risk (HbA1c between 5.7 and 6.4%) for DM2 development.

Besides the impairment in glycemic homeostasis, the occurrence of dyslipidemias secondary to the E2 downfall is common in postmenopausal women [24]. In our study, we did not find any effect of the postmenopause condition on HDL levels. It may be related to the already reported dysfunctional HDL, followed by a compensatory increase in its levels in menopause conditions [25, 26]. Besides, LDL was also affected by menopause, but its levels were higher in women without DM2. These intriguing results may be related to the use of cholesterol-lowering medications, such as statins, which were associated with LDL levels. DM2 also affected triglycerides levels, as already described [27].

Diabetes increases the circulating levels of fatty acids, which can overload the liver. Thus, we evaluated the hepatic function by measuring hepatic enzymes. Although the GOT and GPT were unchanged, menopause enhanced ALF levels independent of DM2, and GGT was affected by both factors. Besides acting as a biomarker of hepatic function, GGT plays a role in intracellular glutathione synthesis and the antioxidant response [28]. Such antioxidant response may be impaired in DM2, which is marked by chronic and low-grade oxidative and inflammatory conditions that follow the metabolic alterations [29].

Accordingly, the postmenopausal women with DM2 presented the highest plasmatic lipoperoxidation levels. Estrogen, mainly 17*β*-estradiol (E2), is a powerful antioxidant [28] by modulating the expression and activity of several antioxidant enzymes. Thus, hypoestrogenism in postmenopause impairs the redox status [2, 30], which is exacerbated in diabetic women. We confirmed the association between lipoperoxidation and DM2 by performing a multivariate regression. Hence, these results show that menopause and DM2 present synergistic effects, and their association potentialize the risk of oxidative damage.

E2 is also a powerful anti-inflammatory hormone, and the lack of it represents a risk for the establishment of a chronic and low-grade inflammatory condition. In our study, menopause isolated and combined with DM2 enhanced the leukometry in a way that postmenopausal women with DM2 had the highest numbers. Leukocytosis mainly represents the enhancement of myeloid cell production and the number of circulating neutrophils and monocytes in senescence [31] and hyperglycemia, which mark the inflammatory status [31, 32]. Thus, the synergistic effects of menopause and DM2 found in our study support that postmenopausal women with DM2 are exposed to an additional risk for cardiovascular outcomes [33].

In agreement with that, the interaction between menopause and DM2 also affected ESR, a routinely used biomarker of inflammation. ESR does not respond quickly to the initial stages of the inflammatory process and remains elevated for longer periods than acute-phase proteins, such as CRP [34]. Interestingly, the CRP levels were not affected by these risk factors. CRP is an acute phase biomarker whose hepatic production and release are induced by IL-6 and TNF-*α*, highly available in menopause [35]. Besides, CRP is not just a biomarker but also a crucial factor in obesity development [36].

Despite being a well-recognized inflammatory biomarker, in our study, CRP was not affected by menopause nor diabetes. Since CRP was described as the strongest factor associated with overweight [37], the lack of responsiveness may be because the women participating in our study did not differ in anthropometric measurements despite the diabetes diagnosis. Thus, it led us to investigate whether HSP72 could be more sensitive to menopause plus diabetes low-grade inflammatory background.

HSP72 plays a fundamental role as a chaperone, anti-inflammatory, antioxidant, and antiapoptotic protein [38], whose expression is regulated by E2 [39, 40]. However, previous studies showed that a short and middle deprivation of E2 does not downregulate HSP72 expression [41, 42], which suggests that the cellular response to stress during hypoestrogenism may depend on the time of postmenopause [43]. We found a curvilinear relationship between eHSP72 levels and the time of menopause, with decreasing values during the first 11 years and increasing values between 12 to 29 years of menopause. Thus, we stratified the postmenopausal women according to the time of menopause and found that the group with longer periods of postmenopause had high eHSP72 levels.

The time of postmenopause is naturally related to the lifespan. Considering that aging impairs the heat shock response, independent of E2 deprivation [43], we analyzed whether the eHSP72 levels in postmenopausal women could be related to their age rather than the time of menopause. Thus, we compared postmenopausal women younger (≤60 years) and older than 60 years old (≥60 years), and the eHSP72 levels did not differ among groups. These data reinforce the responsiveness of eHSP72 to the time of menopause, which may represent an attempt to restore the nitric oxide-HSP72 axis, highly required during estrogen downfall and in postmenopause [3].

In the extracellular medium, the importance of eHSP72 resides in warning signaling [9], immune-regulation, and mediating proinflammatory pathways [44, 45]. Moreover, eHSP72 levels correlate with the severity of atherosclerosis [46] and with the time of diagnosis of DM2 [47]. Until this moment, whether eHSP72 could help mark the complexity of the menopausal period was unclear. We showed that eHSP72 levels are also sensitive to the time of postmenopause and would be a potential biomarker of chronic conditions related to the metabolic impairment present in postmenopause. Besides, considering that the well-recognized inflammatory biomarker CRP [36, 37, 48] was not responsive in our study, we also suggest that in menopause plus diabetes low-grade inflammatory background, the serum eHSP72 levels may be more sensitive and a better biomarker of the preclinical stages.

## 5. Conclusion

The time of menopause enhances the plasmatic eHSP72 levels, which are also sensitive to E2 levels and DM2. Besides, menopause associated with DM2 is marked by an additional risk of systemic oxidative damage. Together, menopause and DM2 present synergistic effects in metabolic, oxidative, and inflammatory parameters and require special attention. Most important, in menopause plus DM2 chronic low-grade inflammatory background, eHSP72 levels can be a potential biomarker and a better alternative of follow-up than well-established biomarkers.

## Figures and Tables

**Table 1:** Anthropometric, metabolic, oxidative, inflammatory, and hepatic parameters from pre- and postmenopausal women, with or without DM2 diagnosis.

Parameters		Premenopausal	Premenopausal with DM2	Postmenopausal	Postmenopausal with DM2	*p* value
Anthropometric parameters	BMI	30.42 ± 4.46	33.94 ± 8.26	31.02 ± 4.96	28.67 ± 6.02	n/s
	AC	101.13 ± 10.31	101.00 ± 14.75	101.90 ± 8.01	95.17 ± 10.87	n/s
	WHR	0.87 ± 0.09	0.92 ± 0.05	0.92 ± 0.06	0.92 ± 0.06	n/s
	BAI	35.48 ± 3.45	39.60 ± 10.90	37.70 ± 5.05	35.27 ± 4.55	n/s
	CI	1.24 ± 0.12	1.30 ± 0.08	1.32 ± 0.08	1.32 ± 0.11	n/s

Metabolic parameters	Gli	92.00 ± 7.58	132.86 ± 25.94^ab^	92.47 ± 8.03	136.92 ± 31.40^ab^	Menopause *p* = 0.6450, diabetes *p* < 0.0001, interaction *p* = 0.7144
	HbA1c	5.40 ± 0.33	6.80 ± 1.10^ab^	5.85 ± 0.33	7.15 ± 1.63^ab^	Menopause *p* = 0.1039, diabetes *p* < 0.0001, interaction *p* = 0.8347
	MEG	108.28 ± 9.48	148.46 ± 31.48^ab^	121.24 ± 9.60	158.50 ± 45.88^ab^	Menopause *p* = 0.1039, diabetes *p* < 0.0001, interaction *p* = 0.8347
	Trig	114.93 ± 33.00	190.71 ± 84.31	128.47 ± 54.56	142.08 ± 66.86	Menopause *p* = 0.2834, diabetes *p* = 0.0077, interaction *p* = 0.0600
	T Col	189.93 ± 47.30	175.86 ± 28.57	214.63 ± 38.56	184.17 ± 31.18	Menopause *p* = 0.1409, diabetes *p* = 0.0486, interaction *p* = 0.4617
	HDL	52.33 ± 9.10	52.57 ± 12.88	52.13 ± 8.55	49.42 ± 9.62	n/s
	LDL	121.6 ± 39.68	85.00 ± 29.25	139.77 ± 31.16	106.33 ± 26.06^b^	Menopause *p* = 0.0366, diabetes *p* = 0.0004, interaction *p* = 0.8645

Hepatic parameters	OGT	24.73 ± 9.96	22.71 ± 7.27	24.27 ± 6.05	25.00 ± 5.31	Menopause *p* = 0.6582, diabetes *p* = 0.7544, interaction *p* = 0.5036
	GPT	20.93 ± 11.18	18.71 ± 6.75	19.97 ± 5.86	22.58 ± 15.00	Menopause *p* = 0.5974, diabetes *p* = 0.9423, interaction *p* = 0.3799
	GGT	24.4 ± 10.05	52.43 ± 27.63^ab^	26.10 ± 10.04	32.17 ± 16.59^c^	Menopause *p* = 0.0247, diabetes *p* < 0.0001, interaction *p* = 0.0084
	ALF	99.00 ± 27.15	87.43 ± 22.29	112.67 ± 34.11	109.25 ± 25.23	Menopausal *p* = 0.0428, diabetes *p* = 0.3856, interaction *p* = 0.6361

Oxidative and inflammatory parameters	MDA	0.02 ± 0.05	0.03 ± 0.04	0.007 ± 0.001	0.155 ± 0.0147^abc^	Menopause *p* = 0.0096, diabetes *p* = 0.0002, interaction *p* = 0.0008
	Leukometry	6.56 ± 1.57	6.21 ± 1.07	6.61 ± 1.58	8.50 ± 2.76^b^	Menopause *p* = 0.0330 diabetes *p* = 0.1538 interaction *p* = 0.0412
	ESR	12.58 ± 10.30	38.86 ± 12.61	31.97 ± 26.13	29.17 ± 26.53	Menopause *p* = 0.4711, diabetes *p* = 0.0843, interaction *p* = 0.0337
	CRP	0.48 ± 0.59	0.77 ± 0.82	0.52 ± 0.47	0.61 ± 0.61	Menopause *p* = 0.7057, diabetes *p* = 0.2545, interaction *p* = 0.5432

**Table 2:** Multivariate regression analysis between eHSP72, age, E2 levels, time of menopause, and DM2 in postmenopausal women.

	Multivariate regression ANOVA		
	*R* ^2^_aj_	*F*	*p*
Variables	0.48	7.945114	
Age			0.5143
E2 levels			0.0018
Time of menopause			0.0152
DM2			0.0090

E2:17-*β* estradiol (pg/mL); DM2: type II diabetes mellitus (absence = 0 and presence = 1); time of menopause and age (years). *R*^2^_aj_: R-adjusted square (*n* = 31).

## Data Availability

The datasets used and/or analyzed during the current study are available from the corresponding author upon reasonable request.
